# SAAFEC-SEQ: A Sequence-Based Method for Predicting the Effect of Single Point Mutations on Protein Thermodynamic Stability

**DOI:** 10.3390/ijms22020606

**Published:** 2021-01-09

**Authors:** Gen Li, Shailesh Kumar Panday, Emil Alexov

**Affiliations:** Department of Physics and Astronomy, Clemson University, Clemson, SC 29634, USA; genl@clemson.edu (G.L.); spanday@clemson.edu (S.K.P.)

**Keywords:** thermodynamics stability, single point mutation, sequence-based, machine learning, web server

## Abstract

Modeling the effect of mutations on protein thermodynamics stability is useful for protein engineering and understanding molecular mechanisms of disease-causing variants. Here, we report a new development of the SAAFEC method, the SAAFEC-SEQ, which is a gradient boosting decision tree machine learning method to predict the change of the folding free energy caused by amino acid substitutions. The method does not require the 3D structure of the corresponding protein, but only its sequence and, thus, can be applied on genome-scale investigations where structural information is very sparse. SAAFEC-SEQ uses physicochemical properties, sequence features, and evolutionary information features to make the predictions. It is shown to consistently outperform all existing state-of-the-art sequence-based methods in both the Pearson correlation coefficient and root-mean-squared-error parameters as benchmarked on several independent datasets. The SAAFEC-SEQ has been implemented into a web server and is available as stand-alone code that can be downloaded and embedded into other researchers’ code.

## 1. Introduction

Proteins carry their function by adopting a particular 3D structure and the ability to fold into a 3D structure is governed by the folding free energy. Thus, assessing the effect of amino acid mutations on the folding free energy (ΔΔG) is essential for evaluating the effect of mutations on structural stability of proteins [1,2]. While experimental investigations are preferred, they are too expensive and time consuming to be applied on a large number of cases [3,4]. Due to that, computational approaches that can accurately predict the change of the folding free energy (ΔΔG) caused by mutations are highly desirable [5,6]. Such an urgency for developing accurate methods for predicting ΔΔG stems from plausible applications in protein engineering, personalized medicine, and precision diagnostics [7,8,9]. It is speculated that many genetic disorders are caused by missense mutations that alter the wild type protein stability [10,11]. Furthermore, significant correlation was found between the magnitude of the folding free energy change caused by mutations and propensity for the mutations to be pathogenic [12].

Such a need for methods for predicting ΔΔG caused by mutations resulted in development of many methods [13,14]. These methods can be broadly grouped into two categories: structure-based and sequence-based approaches. Most of the methods are structure-based, which include FoldX [15], PoPMuSiC [16], mCSM [17], STRUM [18], SDM2 [19], and SAAFEC [20]. Of a particular interest are methods that do not rely on a 3D structure of the corresponding wild type protein, but utilize only sequence information and, thus, are applicable to genome-scale investigations (note that only about 0.2% of the proteins in UniProt have 3D structure experimentally available [18]). The sequence-based methods take the amino acid sequence of proteins and apply machine learning techniques to predict changes in the stability. The performance of these sequence-based methods was assessed by Khan et al. [21] and it was shown that some sequence-based methods reached and even exceeded the performance of structure-based methods. Below, we outline some of the prominent sequence-based methods. The I-Mutant2.0 [22] is a model based on sequence features alone and reported to achieve a Pearson correlation coefficient (PCC) of 0.62 as benchmarked on the S2648 dataset. The (Evolutionary, Amino acid, and Structural Encodings with Multiple Models) EASE-MM [23] method is based on the predicted secondary structural elements, evolutionary information, and physicochemical properties and is reported to achieve a PCC of 0.56 as benchmarked on the S1676 dataset. The (Impact of Non-synonymous mutations on Protein Stability) INPS [24] method also takes the mutability index, evolutionary information, and physicochemical properties as descriptors to predict ΔΔG. More recently, the BoostDDG [25] method was reported and it uses predicted structural features, evolutionary information, and physicochemical properties, and, in a cross-validation test, it achieved 0.54 on the S2815 dataset. The list can be extended to mention other sequence-based methods, but their overall performance is similar to those outlined above. Here, we would like to clarify that the list of methods is neither exhaustive nor the provided PCC should be considered for ranking the methods. This is because they were trained and tested on different datasets, and were shown to be very sensitive with respect to the dataset used for benchmarking.

Another important aspect of the method performances is that they were shown to deliver better predictions for destabilizing mutations as compared with the predictions for stabilizing mutations [26,27]. This was attributed to the training data (typically taken from the ProTherm database [28]), which contains much more experimentally determined destabilizing ΔΔGs. This asymmetry of the training database prompted some researchers to consider reverse mutations by simply changing the sign of ΔΔGs [25]. Such an approach definitely makes the database symmetrical, but results in an artificial increase of the data points (because reverse ΔΔGs contain the same information as forward ΔΔGs). For these considerations, we do not involve reverse mutations in our work.

Here, we report a new version of Single Amino Acid Folding free Energy Changes (SAAFEC) [20], the SAAFEC-SEQ, which is a sequence-based method and replaces the old SAAFEC. Compared with the previous SAAFEC, the new SAAFEC-SEQ does not use energy terms (van der Waals, electrostatics, etc.) calculated from the 3D structure of the corresponding protein. Instead, it utilizes knowledge-based terms and evolutionary information and does not require a 3D structure of the protein. The method uses a gradient boosting decision tree machine learning algorithm with features as physicochemical properties of the mutation site, sequence features, and evolutionary information to predict the change of folding free energy resulting from a single amino acid mutation. The method is developed by performing 100 runs of five-fold cross-validations, and it is shown to achieve a Pearson correlation coefficient (PCC) of 0.74 and a mean-squared-error (MSE) of 0.95 kcal/mol as benchmarked against 2648 experimental ΔΔGs taken from the ProTherm [28] database. Further SAAFEC-SEQ was validated on 350 mutations randomly chosen from the S2648 dataset to confirm its performance and achieved a PCC of 0.78. Furthermore, we tested SAAFEC-SEQ on three independent blind sets (S276 [29], *p*53 [17], CAGI5 [30] datasets, respectively), and showed that SAAFEC-SEQ performs better compared with other sequence-based methods. Moreover, SAAFEC-SEQ, which is a sequence-based method, achieved a performance comparable to or better than the structure-based methods. The SAAFEC-SEQ has been implemented into a user-friendly webserver and a standalone code are freely available at http://compbio.clemson.edu/SAAFEC-SEQ/index.php.

## 2. Results

### 2.1. SAAFEC-SEQ Training and Testing

**Figure 1:**
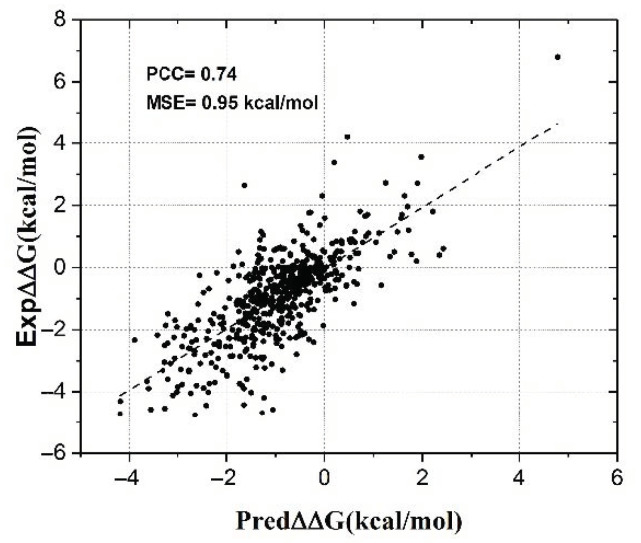
SAAFEC-SEQ predicted ΔΔG against experimental ΔΔG in case of 20% of mutations as a test set.

We trained SAAFEC-SEQ on a frequently used dataset containing experimental ΔΔG of 2648 single point mutations from 131 different proteins, which were taken from ProThem [28]. In a five-fold cross-validation, our model shows a correlation of 0.74 and MSE of 0.95 kcal/mol when tested on 20% of the dataset. As we can see from Figure 1, the distribution of predicted ΔΔGs using SAAFEC-SEQ is remarkably similar to corresponding experimental ΔΔGs.

### 2.2. Feature Importance Analysis

**Figure 2:**
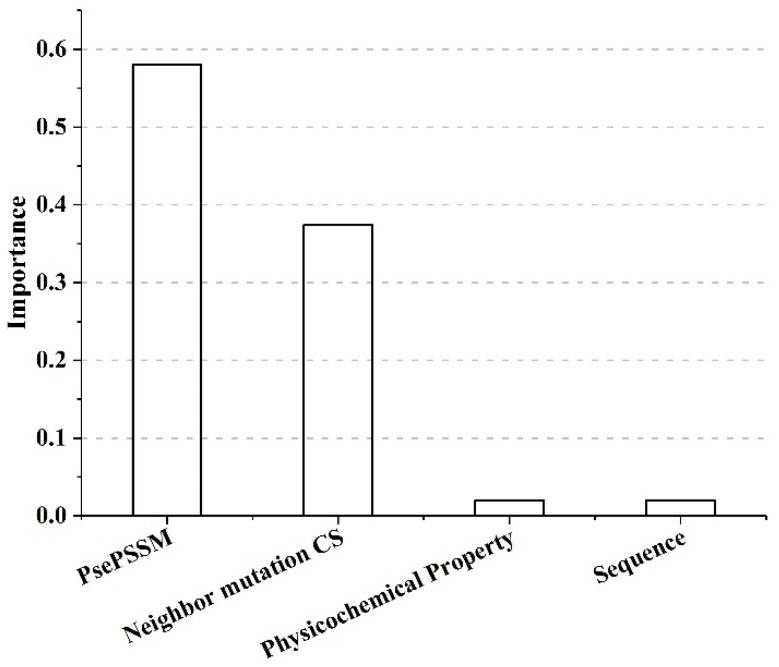
Importance level of each feature selected for SAAFEC-SEQ.

SAAFEC-SEQ uses four group features to discriminate the stability changes caused by single point missense mutations. To evaluate the feature’s importance, we used the decision tree algorithm implemented in python package XGBoost [31], which is used for the training model and feature importance is computed using gradient boosting. It involves computing the amount of performance measure improvement due to each attribute split point weighted by the number of observations the node is responsible for in a single decision tree. Then, the feature importance across all the decision trees within the model are averaged for each attribute [31]. Figure 2 shows the importance level of each group feature and reveals that PsePSSM of the protein and neighbor mutation conservation scores (neighbor mutation CS) are the two most important group features in our model. These two features capture the evolutionary conservation of a given amino acid at the mutation site as well as of the surrounding of the mutation site and its change upon mutation. PSSM has already been established for providing crucial information in hot-spot [32] binding site [33] predictions and thermodynamics stability predictions [18,23,24,25,29]. The third highest contributing group feature is physicochemical property. The next important feature is the sequence neighbor, where we took into account 10 amino acids near the mutation site, according to the primary sequence. The sequence neighbor feature captures the influence of neighboring amino acid residues on the mutation site. We also tested the ability of the single group features, and the result showed the efficiency of PsePSSM and neighbor mutation CS to predict the protein stability (Table S1).

### 2.3. Comparison of SAAFEC-SEQ Performance with Other Methods

To assess the ability of the SAAFEC-SEQ to predict the effect of mutations on protein stability, we designed an extensive series of comparative experiments with other existing state-of-the-art methods, including PoPMuSiC [16], mCSM [17], DUET [34], STRUM [18], SDM2 [19], I-Mutant2.0 [22], INPS [24], EASE-MM [23], and BoostDDG [25]. Among them, the last four are sequence-based and others are structure-based methods. We used a dataset of 2648 mutations (S2648) to develop our method and estimate its performance using a five-fold cross-validation. Next, we employed three independent datasets of 350, 276, and 42 mutations and the corresponding datasets are S350 [16], S276 [29], and *p*53 [17], respectively. Finally, we applied SAAFEC-SEQ on the PTEN [35] and TPMT [36] datasets to further benchmark it.

#### 2.3.1. Comparison of SAAFEC-SEQ Performance with Other Methods on S2648 and S350 Datasets

Table 1 lists the PCC and MSE between predicted and experimental ΔΔGs obtained by different methods. Note that the prediction results of other methods are collected from literature [16,17,18,19,22,23,24,25,29,34]. The first comparison is made on a widely used training set of mutations, the S2648 dataset [16], and in a five-fold cross-validation test performed 100 times using SAAFEC-SEQ reaches the average correlation coefficient 0.75, which is the second-highest among all methods and significantly outperforms the rest of the sequence-based methods (Table 1). Furthermore, the MSE value obtained by SAAFEC-SEQ (MSE = 0.95 kcal/mol) is also lower than other predictions except for the STRUM method (second and third columns). If we only compare with sequence-based methods, not only is the PCC the highest but also the MSE is the lowest among the sequence-based methods (Table 1) (Note that we exclude BoostDDG and EASE-MM from Table 1 because they were trained on different training datasets. However, for reference, their PCCs are reported to be only 0.54 and 0.56, respectively). To check for plausible overfitting due to the presence of mutations corresponding to the same or homolog proteins both in a training and testing set [37,38], we generated a more stringent test by randomly selecting 20% of S2648 as a testing set and assuring that each test case have a sequence identity less than 30% to any proteins in the training set. Benchmarking SAAFEC-SEQ on this test set resulted in PCC of 0.55 and MSE of 1.34 kcal/mol, which is still acceptable.

The S350 dataset is a randomly selected subset from the S2648 dataset, which most of the methods have used for benchmarking. Similarly to S2648, we trained SAAFEC-SEQ on the 2298 ΔΔGs (after removing S350 from S2648) and tested it on S350. As shown in Table 1 (fifth and sixth columns), the performance of SAAFEC-SEQ (PCC = 0.78) on S350 is almost the same as the highest method (STRUM, PCC = 0.79), and the MSE is the lowest among all predictors.

#### 2.3.2. Blind Test on Two Datasets: *p*53 and S276

The *p*53 protein is a tumor suppressor that plays a crucial role in the cell cycle, apoptosis, and genomic stability [39]. Mutations on *p*53 are found in approximately half of human cancers [40]. Pires et al. [17] created the *p*53 database and used it as a benchmark to test predictors. In this dataset, there are 42 single missense mutations of which none appears in our training dataset.

Table 2 lists the results of ΔΔG predictions by 10 different methods where structured-based predictors: I-Mutant2.0, SDM2, INPS3D, STRUM, mCSM, DUET, and PoPMuSiC used the published crystal structure (PDBID: 2OCJ) and the rest of the methods used the sequence of *p*53. Table 2 shows that the stability changes predicted by SAAFEC-SEQ result in the second highest correlation with the experimental values (PCC = 0.70). If we only compare with sequence-based methods, SAAFEC-SEQ is better than others, especially BoostDDG, EASE-MM, and I-Mutant2.0. Similarly, SAAFEC-SEQ MSE is one of the lowest.

We also tested SAAFEC-SEQ on the S276 dataset, which consists of 276 single mutations in 37 different proteins. This blind set was collected from Cao’s study [29] and it was not used in our training set or validation. Since the previous studies [25,29] reported a mean absolute error (MAE), we also applied MAE as a measurement of the performance. Table 2 represents a prediction comparison in the form of PCC and MAE obtained using different ΔΔG predictors along with SAAFEC-SEQ. The PCC and MAE values, achieved by other methods, are taken from a previous paper [25]. As Table 2 shows (fourth and fifth columns), BoostDDG achieves the highest PCC of 0.51, although a PCC of 0.51 is not impressive at all. SAAFEC-SEQ PCC is not impressive either (PCC = 0.46). However, other methods also do not perform well. However, SAAFEC-SEQ MAE = 0.89 is the second-lowest.

#### 2.3.3. Performance on the Independent CAGI Dataset

**Figure 3:**
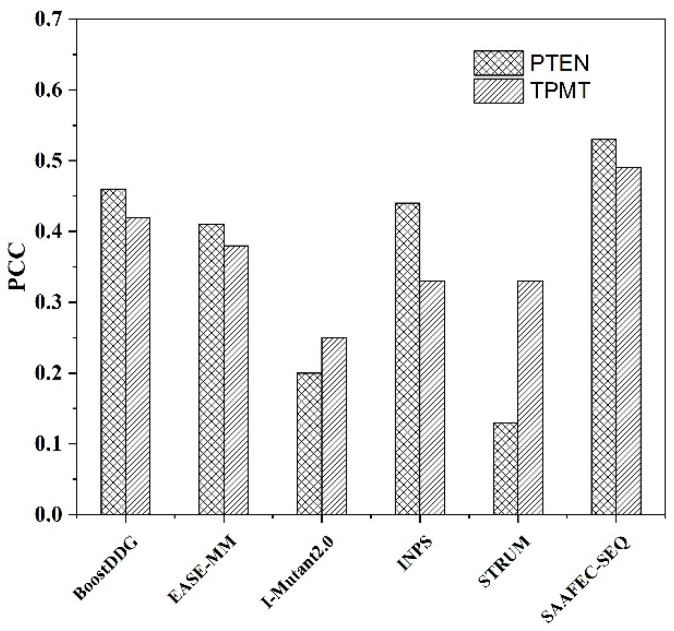
Performance comparison of SAAFEC-SEQ with other existing sequence-based methods on PTEN and TPMT datasets.

SAAFEC-SEQ was further tested against the Critical Assessment of Genome Interpretation 5 challenge (CAGI 5) [30], which is composed of 7363 experimentally determined effects of mutations in two proteins: Phosphatase and TEnsin Homolog (PTEN, 3736 mutations) and Thiopurine S-methyl transferase (TPMT, 3627 mutations) [25]. The mutants were subjected to deep mutational scans [41] to calculate the stability score of the mutation. Three scores were defined: score between 0 and 1 denotes an unstable protein, score of 1 denotes that the mutant is stable as the wild type, and >1 denotes that the mutant is more stable than the wild type. Because of the lack of PTEN and TPMT crystal structures, we used the full-length sequence of the two proteins (Uniprot ID P60484 and P51580) to compare SAAFEC-SEQ with the other sequence-based methods: BoostDDG, EASE-MM, I-Mutant2.0, and INPS. STRUM is a structure-based predictor but can take input protein sequences and uses predicted structures. Figure 3 represents a prediction comparison in the form of PCC obtained using different sequence-based ΔΔG predictors along with SAAFEC-SEQ. The SAAFEC-SEQ achieved Pearson correlations of 0.53 and 0.49 for PTEN and TPMT, respectively. None of the other sequence-based methods achieved a correlation PCC over 0.46. Among these methods, BoostDDG, EASE-MM, and INPS have similar results, whereas I-Mutant2.0 and STRUM generated poor results. One possible reason for the dissatisfactory results of STRUM on PTEN is that STRUM requires a reliable 3D model to predict when there is no experimental structure [25]. The trend also can be observed in *p*53 blind tests (Table 2) where STRUM uses a predicted structure instead of an experimental structure. Furthermore, the SAAFEC-SEQ results are significantly different from other methods by a significance test (Fish-z test). The results of SAAFEC-SEQ on PTEN and TPMT datasets indicate it is more accurate than other sequence-based methods and shows its ability to predict the protein stability changes upon mutations without using a 3D structure. There is another protein stability prediction challenge (Frataxin challenge [42]) in CAGI 5, which consists of eight single point mutations in the Frataxin protein. Applying SAAFEC-SEQ on this case resulted in a PCC of 0.72 (Table S2).

### 2.4. Webserver

The SAAFEC-SEQ user-friendly web server is freely accessible at http://compbio.clemson.edu/SAAFEC-SEQ/index.php. It is hosted on the Palmetto cluster for processing the user’s input. Three alternatives are available: (i) Predict the effect of a single mutation specified by the user in the given boxes. User needs to provide a FASTA sequence of the protein by uploading the file in the FASTA format or by inputting the sequence in the appropriate box. In this way, users can submit a single job. (ii) A single file containing the list of point mutations that are relative to the sequence, in addition to uploading or inputting sequences of protein in a FASTA format, users need to upload a mutations list file. (iii) Users can also directly download the SAAFEC-SEQ code from our webpage. A readme file will also be included, which will guide the user on how to use the code.

## 3. Discussion

Here, we reported a new algorithm and a webserver (http://compbio.clemson.edu/SAAFEC-SEQ/index.php), known as the SAAFEC-SEQ method, which only uses sequence information to predict ΔΔG. We benchmarked the SAAFEC-SEQ against 2648 experimental data-points and achieved a correlation coefficient of 0.75, which is the best among all existing sequence-based predictors. Furthermore, the SAAFEC-SEQ comes as a stand-alone that can be downloaded and implemented as third party software. Considering the parallel-computing capability of modern computers, we expect that the SAAFEC-SEQ method will be a useful tool for genome-scale investigations.

As any machine learning method, the performance of SAAFEC-SEQ depends on the training dataset and selection of features. The training dataset is a ProTherm database [28], which is a collection of experimentally measured folding-free energy of wild type and mutant proteins. In the vast majority of cases, the folding-free energy of the mutants was found to be less favorable than of a wild type, and, thus, the dataset is biased toward de-stabilizing mutations. Series of works [26,43,44,45] were devoted on this topic and suggested that the training (and testing) dataset should have a similar number of cases of stabilizing and de-stabilizing mutations. While this is understandable from the point-of-view of statistics, we argue that this should not be necessarily applied in developing machine learning predictors of protein stability changes caused by mutations (especially for predictors that use only sequence information as SAAFEC-SEQ). The reason for such a claim is that the wild type protein sequences evolved to adopt a particular 3D structure and, thus, they are nearly optimized with respect to folding free energy. Thus, most random mutations are expected to destabilize the corresponding protein (as seen in the ProTherm database), despite that, in principle, one can engineer more stable variants [28]. Therefore, de-stabilizing mutations will be always much more than stabilizing ones. Furthermore, the SAAFEC-SEQ uses only sequence information (as PSSM, etc.) to deliver the predictions and, thus, is biased toward the natural selection that the wild type structure in nearly folding free energy optimized and most of mutations are expected to destabilize the corresponding protein. As shown in Table S6, SAAFEC-SEQ predictions on reverse mutations are not impressive. 

## 4. Materials and Methods 

### 4.1. Dataset Collection

We used several different datasets to develop, validate, and independently test the SAAFEC-SEQ method. These datasets contain experimental thermodynamic information for wild type and mutant proteins, including the change in Gibbs free energy (ΔΔG). The following datasets contain only a single chain protein and single point missense mutations.

S2648. This is our training and test dataset, the S2648, collected from the ProTherm database [28], including 2648 unique single point missense entries in 131 different proteins and the corresponding ΔΔGs.

S350. This is our validation dataset. To compare with other methods, we used the same validation dataset used by other developers [16,17,18,19], which contains 350 mutations (taken from 67 different proteins) randomly selected from S2648. 

S276. This blind data set was collected from Cao’s et al. work [29], which includes 276 unique single point missense entries in 37 different proteins. None of them is in the training or validation set.

*p*53. This is the second blind dataset. We used a dataset of 42 single point missense mutations within the DNA binding domain of the tumor suppressor protein *p*53, which thermodynamic effects have been experimentally determined [46,47,48]. As in the previous case, none of them appeared in our training set.

PTEN and TPMT. For the third blind data set, we collected two independent datasets for the phosphatase and tensin homologue (PTEN) and thiopurine S-methyl transferase (TPMT) proteins from the Critical Assessment of Genome Interpretation (CAGI) challenge [30]. It can be downloaded from https://genomeinterpretation.org/content/predict-effect-missense-mutations-pten-and-tpmt-protein-stability. We removed mutations with an unknown amino acid “X” (both in wild type and mutant), and then kept a total of 7363 missense mutations for the PTEN (3736) and TPMT (3627) proteins.

### 4.2. Sequence-Based Features

#### 4.2.1. Pseudo-Position Specific Scoring Matrix (PsePSSM)

In order to consider the sequence-order property of the amino acid residues in the protein sequence, we used a Pseudo-Position Specific Scoring Matrix (PsePSSM) [49] as input features.

(1) $$P_{PsePSSM}={\left(\bar{P_{1}},\bar{P_{2}},\cdots ,\bar{P_{20}},\phi_{1}^{1},\phi_{2}^{1},\cdots ,\phi_{20}^{1},\cdots ,\phi_{1}^{\varphi },\phi_{2}^{\varphi },\cdots ,\phi_{20}^{\varphi }\right)}^{T}$$

where $\bar{P_{j}}$ is the average of each column in PSSM, and $\phi_{j}^{\varphi }$ can be expressed as follows.

(2) $$\phi_{j}^{\varphi }=\frac{1}{L-\varphi }\overset{L-\varphi }{\underset{i=1}{\sum }}{\left(P_{i,j}-P_{\left(i+\varphi \right),j}\right)}^{2}(j=1,2,\cdots 20;0<\varphi <L)$$

Using this equation, we can get the 20 + 20 × *φ* dimension feature vector, and $\phi_{j}^{\varphi }$ indicates the order property of the protein. In this work, *φ* was set as 7, which can have a significant influence on prediction (Table S3). A more detailed process can be found in the Supplementary Information.

#### 4.2.2. Neighbor Mutation Conservation Scores

To reflect the evolutionary information near the mutation site, we considered a stretch of residues, XXXCXXX, in which C was the mutation site and X referred to the neighboring amino acids. We selected the rows belonging to the mutation site and neighbors from PSSM to obtain 20 × 7 conservation score features (Table S4).

#### 4.2.3. Sequence Neighbors Feature

We selected five amino acids from both the left and right of the mutation site as sequence information. There could be 20 possibilities of each label that represent 20 different amino acids.

#### 4.2.4. Physicochemical Properties Feature

We used nine physicochemical properties related to a mutation site: net volume, net hydrophobicity, mutation type, net flexibility, chemical property, size, polarity, hydrogen bond, and label hydrophobicity. Detailed information could be found in our published papers [50]. 

#### 4.2.5. Regression Model Development

The SAAFEC-SEQ model was built and trained by using the XGBoost python version, which has shown the advantage to overcome the over-fitting effect compared with many other machine learning methods. GridSearchCV [51] was used to search the hyper-parameters of the XGBoost model. The hyper-parameter settings for the model, which is used to train SAAFEC-SEQ, are provided in Table S5. For predicting ΔΔG upon a given mutation, we developed a regression model by using knowledge-based features, representing evolutionary information, and a physicochemical environment surrounding the mutation site. In order to build a reliable and robust model, we performed five-fold cross-validation 100 times. Selection of the training and test sets were repeated 100 times randomly, and average PCC and MSE are taken into account. We trained our model against 80% of the 2648 mutations present in our compiled dataset and tested against the remaining 20% data.

## Figures and Tables

**Table 1:** Comparison of different methods on the S2648 and S350 datasets.

Method	S2648		S350	
	PCC	MSE ^a^	PCC	MSE ^a^
I-Mutant2.0	0.71 *	1.69	0.29 *	2.72
I-Mutant2.0 ^b^	0.62 *	2.10	-	-
INPS ^b^	0.52 *	1.59	0.68 *	1.59
INPS3D	0.58 *	1.44	0.72	1.32
STRUM	0.77	0.88	0.79	0.96
DUET	0.74	0.96	0.71 *	1.28
SDM2	0.48 *	2.13	0.61 *	1.66
mCSM	0.69 *	1.14	0.73	1.17
PoPMuSiC	0.61 *	1.37	0.67 *	2.79
SAAFEC-SEQ ^b^	0.75	0.95	0.78	0.93

^a^ MSE of ΔΔG prediction in kcal/mol. * *p* < 0.05. ^b^ Sequence-based method. ‘-’ indicates data is unavailable.

**Table 2:** Comparison of different methods on the *p*53 and S276 datasets.

Method	*p*53		S276	
	PCC	MSE ^a^	PCC	MAE ^a^
BoostDDG ^b^	0.49	3.57	0.51	0.78
EASE-MM ^b^	0.59	2.68	0.40	0.91
I-Mutant2.0 ^b^	0.35 *	3.05	0.39	1.08
I-Mutant2.0	0.47	2.58	0.45	0.91
DUET	0.68	1.93	0.44	0.92
INPS ^b^	0.69	2.29	0.47	0.89
INPS3D	0.76	1.84	0.49	0.87
STRUM	0.69	1.79	0.44 ^d^	0.91 ^d^
STRUM ^c^	0.47	3.29	0.45	0.88
SDM2	0.68	2.43	0.48	1.02
mCSM	0.67	1.96	0.47	0.90
PoPMuSiC	0.56	2.50	0.44	0.91
SAAFEC-SEQ ^b^	0.70	1.91	0.46	0.87

^a^ MSE/MAE of ΔΔG predictions in kcal/mol. * *p* < 0.05. ^b^ Sequence-based method. ^c^ STRUM with predicted 3D structures. ^d^ Results do not include 16 mutants within the PDB ID 1FC1 structure because the runs failed.

## Data Availability

Data is available at http://compbio.clemson.edu.

## Supplementary Materials

Supplementary Materials can be found at https://www.mdpi.com/1422-0067/22/2/606/s1.

## Abbreviations

CS	Conservation scores
PCC	Pearson correlation coefficient
PsePSSM	Pseudo-Position Specific Scoring Matrix
MSE	Mean squared error
PTEN	Phosphatase and tensin homologue
TPMT	Thiopurine S-methyl transferase
