# Supplementary material for: SAAFEC-SEQ: A Sequence-Based Method for Predicting the Effect of Single Point Mutations on Protein Thermodynamic Stability
Source: Int J Mol Sci. 2021 Jan 9;22(2):606. doi: 10.3390/ijms22020606 (PMC7827184; doi:10.3390/ijms22020606)
Supplement: Supplementary file 1 [file ijms-22-00606-s001.pdf]

SAAFEC-SEQ: A Sequence-based Method for Predicting the Effect of Single Point Mutations on Protein Thermodynamic Stability

Gen Li [1], Shailesh Kumar Pandey[1] and Emil Alexov [1,*]

[1] Department of Physics and Astronomy, Clemson University, Clemson, SC 29634, USA; genl@clemson.edu (G.L.); spanday@clemson.edu (S. Pandey)

* Correspondence: ealexov@clemson.edu

Table S1. PCC between experimental ΔΔG and ΔΔG predicted by using each feature groups.

Feature Groups	Performance	
	PCC	MSE
Physicochemical Property	0.39	1.87
Sequence	0.43	1.81
Neighbor mutation CS	0.50	1.63
PsePSSM	0.51	1.73

Table S2. Comparison of different methods on the Frataxin challenge.

Method	Performance	
	PCC	MSE
SAAFEC-SEQ	0.72	11.62
I-Mutant2.0	0.75	10.82
INPS	0.64	10.85
EASE-MM	0.85	8.75
BoostDDG	0.64	11.48
STRUM	0.86	10.82

Table S3. Effect of selecting different window sizes of φ on the prediction results in case of 20% of mutations as test set.

Windows	Performance	
	PCC	MSE
0	0.65	1.09
1	0.67	1.15
2	0.69	1.12
3	0.69	1.03
4	0.69	1.07
5	0.71	1.03
6	0.72	0.97
7	0.74	0.95
8	0.74	0.98
9	0.74	0.96
10	0.73	0.95
11	0.71	1.02

Table S4. Effect of selecting different PSSM windows size on the prediction results in case of 20% of mutations as test set.

Windows	Performance	
	PCC	MSE
0	0.66	1.23
3	0.69	1.08
5	0.72	1.05
7	0.74	0.95
9	0.73	0.99
11	0.71	1.04

Table S5. The hyper-parameter settings for the model used to train SAAFEC-SEQ.

Parameter	Range	Setting
n_estimators	10-2500	520
max_depth	2-14	5
min_child_weight	1-10	8
min_samples_leaf	2-10	2
min_samples_split	2-12	3
subsample	0.1-1.0	0.95
max_features	1-11	1
gamma	0-0.9	0.6
colsample_bytree	0.1-0.9	0.2
learning_rate	0.001-0.9	0.05
reg_alpha	0.01-3.0	1.41
reg_lambda	0.01-3.0	2.91

Table S6. Performance of direct and inverse mutations belonging to the dataset Ssym

Method	PCC_{dir}	MSE_{dir}	PCC_{inv}	MSE_{inv}
SAAFEC-SEQ	0.75	1.08	-0.43	7.22

Pseudo-Position Specific Scoring Matrix (PsePSSM)

PSSM is one of the most important features used in protein-protein binding predictors. To reflect the evolutionary information, we used protein sequence as the input to search and align homogenous sequences from NCBI non-redundant (nr) protein database (https://ftp.ncbi.nlm.nih.gov/blast/db/) using the PSI-BLAST program[1] with 3 iterations and a cutoff E-value 0.001, then the PSSM was constructed through a

multiple sequence alignment of the highest-scoring hits. As a result, we obtained the L × 20 PSSM for each protein sequence, where L is a given length of each protein sequence. Each row of the PSSM matrix represents the log-likelihood score for amino acid substitutions at the corresponding positions in the input sequence.

$$P_{PSSM} = \begin{pmatrix} P_{1,1} & P_{1,2} \ldots & P_{1,20} \\ \vdots & \vdots \quad \vdots & \vdots \\ P_{i,1} & P_{i,2} \;\cdots & P_{i,20} \\ \vdots & \vdots \quad \ddots & \vdots \\ P_{L,1} & P_{L,1} & P_{L,20} \end{pmatrix} \tag{1}$$

where $P_{i,j}$ represents the score of the amino acid residue in the *i*-th position of the protein sequence being changed to amino acid type *j* during the evolution process.

All values in PSSM of each protein sequence are normalized to be between 0 and 1 by sigmoid function:

$$f(x) = 1/(1 + e^{x}) \tag{2}$$

where x is the original value of PSSM.

Protein sequences of different size have different length of PSSM. To make the PSSM descriptor become a size-uniform matrix, one approach is to represent a protein sample P by

$$\bar{P}_{PSSM} = (\overline{P_1}, \overline{P_2}, \cdots, \overline{P_{20}})^{T} \tag{3}$$

where

$$\bar{P}_j = \frac{1}{L}\sum_{i=1}^{L} P_{i,j} \ \ (\text{j} = 1,2, \cdots 20) \tag{4}$$

and $\bar{P}_j$ is the composition of the amino acid type *j* in the PSSM and represents the average score of the amino acid residues in the protein P being mutated to amino acid

type j during the evolution process. Using this method, we obtained 20 uniform average conservation scores for each input protein sequence.

However, this method only considers the average conservation score of the amino acid residues in the protein sequences changed to the type j amino acid, without considering the sequence-order property of the amino acid residues in the protein sequence. To avoid this, Shen [2] proposed a method named Pseudo-Position Specific Scoring Matrix (PsePSSM) to solve the problem of extracting feature vectors in protein sequences.

Use the following formula to obtain PsePSSM:

$$P_{PsePSSM} = (\overline{P_1}, \overline{P_2}, \cdots, \overline{P_{20}}, \phi_1^1, \phi_2^1, \cdots, \phi_{20}^1, \cdots, \phi_1^{\varphi}, \phi_2^{\varphi}, \cdots, \phi_{20}^{\varphi})^T \quad (5)$$

where $\bar{P}_j$ can be obtained from equation (3), and ϕ_j^{φ} can be expressed as follows:

$$\phi_j^{\varphi} = \frac{1}{L-\varphi}\sum_{i=1}^{L-\varphi}\left(P_{i,j} - P_{(i+\varphi),j}\right)^2 \ (j = 1,2,\cdots 20; 0 < \varphi < L) \quad (6)$$

Using this equation, we can get 20 + 20 × φ dimension feature vector, ϕ_j^{φ} indicates the order property of the protein. In this work, φ was set as 7 which can get a significant influence on prediction.

Reference

1. C. Camacho, Coulouris, G., Avagyan, V., Ma, N., Papadopoulos, J., Bealer, K. and Madden, T. L. BLAST+: architecture and applications. *BMC Bioinformatics* **2009**, *10*, 421.
2. H. B. Shen and Chou, K. C. Nuc-PLoc: a new web-server for predicting protein subnuclear localization by fusing PseAA composition and PsePSSM. *Protein Eng. Des. Sel.* **2007**, *20*, 561-567.
